# A truncated nuclear norm and graph-Laplacian regularized low-rank representation method for tumor clustering and gene selection

**DOI:** 10.1186/s12859-021-04333-y

**Published:** 2022-01-20

**Authors:** Qi Liu

**Affiliations:** College of Computer Science and Engineering, Shandong University of Science and Technology, Qingdao, China

**Keywords:** Low-rank representation, Graph-Laplacian, Truncated nuclear norm, Clustering, Gene selection

## Abstract

**Background:**

Clustering and feature selection act major roles in many communities. As a matrix factorization, Low-Rank Representation (LRR) has attracted lots of attentions in clustering and feature selection, but sometimes its performance is frustrated when the data samples are insufficient or contain a lot of noise.

**Results:**

To address this drawback, a novel LRR model named TGLRR is proposed by integrating the truncated nuclear norm with graph-Laplacian. Different from the nuclear norm minimizing all singular values, the truncated nuclear norm only minimizes some smallest singular values, which can dispel the harm of shrinkage of the leading singular values. Finally, an efficient algorithm based on Linearized Alternating Direction with Adaptive Penalty is applied to resolving the optimization problem.

**Conclusions:**

The results show that the TGLRR method exceeds the existing state-of-the-art methods in aspect of tumor clustering and gene selection on integrated gene expression data.

## Background

In most countries, cancer is the first or second cause of death [1]]. Thus, it is a hot topic to prevent and cure cancer effectively in the medical field. Genes can regulate critical movements of organisms, even including the emergence of cancer [2]. As the improvement of gene sequencing technology, plenty of genomic data are available, which is conducive to researching the pathogenesis of cancer [3]. However, the majority of genomic data have the features of high dimension and small sample, which hinders the advances in medicine studies [4, 5]. Evidently, data dimension reduction acts a momentous role in the process of genomic data analysis.

Data dimension reduction aims to get a significant low-dimensional representation of high-dimensional data, remove redundant features and prevent overfitting. Therefore, it has achieved great successes in many areas, such as characteristic gene selection [6], image analysis [7], and text documents [8]. Principal Component Analysis (PCA) is one of the most classic linear dimension reduction methods [9]. Based on the high efficiency of PCA, it has been widely used on different kinds of data and developed in many fields [5, 10, 11]. To boost the robustness of PCA, Candès et al*.* developed a new PCA in [10], called Robust PCA (RPCA), which is exploited for background modeling from video and analyzing face images. Moreover, in [5], RPCA is exploited for discovering differentially expressed genes by Liu et al. Though, the above PCA methods all have obtained excellent results, the performance of these methods is corrupted with the noisy observation data.

In [12], Wright et al*.* introduced a low-rank matrices recovery approach for removing the noise of data. Then, PCA is applied to the low-rank matrix. The robustness of data processing is enhanced significantly through the approach in [12].

The experimental data **X** are usually obtained from a union of multiple subspaces ${\text{S}} = \sum {{\text{S}}_{1} ,{\text{S}}_{2} , \ldots ,{\text{S}}_{k} }$ rather than a single space, where ${\text{S}}_{i}$ indicates low-dimensional space hidden in high-dimensional space [13–15]. Since these methods related to PCA prefer to research the data obtained from a single low-dimensional space, Liu et al*.* proposed a low-rank representation (LRR) model that can excavate the global distribution between data points to study **X** [16]. LRR strives to look for the lowest rank matrix representation about original data and has got brilliant results in several applications [16, 17]. However, LRR still exists a few shortages, for instance it cannot reveal the local manifold structures of data obtained from a non-linear low-dimensional manifold. Joyfully, various manifold learning models have been put forward, such as ISOMAP [18], Laplacian Eigenmap (LE) [19], Locally Linear Embedding (LLE) [20], and graph-Laplacian regularization [21].

A graph-Laplacian regularized LRR (LLRR) model [14] was developed, which introduces the graph regularization into LRR. In LLRR model, the useful rules hiding among the data points including the global geometric structure and the internal similarity information are all seized. LLRR only exploits one view of data, *i.e.* data manifold for data analysis. Contrasted with LLRR, Latent LRR (LatLRR) model adds another view, *i.e.* feature manifold to do image processing [22]. For solving these minimization problems of LRR, LLRR and LatLRR, the common point is to use the nuclear norm to approximate the rank operator. Given a data matrix **X**, the nuclear norm means that the sum of all singular values belonging to **X**. Since the nuclear norm minimizes the sum of all singular values for accomplishing the minimization problem, all non-zero singular values have different influences for the rank [23]. Thus, the nuclear norm maybe not the best way to approximate the rank of the matrix. To better approximate the rank and handle the non-convex optimizing problems, the truncated nuclear norm (TNN) was proposed in [24] and attracted much attention [13, 23, 25, 26]. The TNN that is the sum of few smallest singular values of a matrix can dispel the harm of shrinkage of the leading singular values, so it may be a more robust regularization to get the rank of a matrix than the nuclear norm.

To strengthen the efficiency and robustness of the model, in our paper, a novel LRR method is developed, named Truncated nuclear norm and graph-Laplacian regularized Low-Rank Representation model (TGLRR). In the objective function of TGLRR, the nuclear norm is replaced by the TNN for reaching the robust approximation of rank function, a graph-Laplacian regularization is imposed to find the local manifold structure, and the *L*_1_-norm is used for realizing the sparse constraints of outliers. The main contributions of our paper are showed as follows. Firstly, compared with the popular LRR model regularized by the nuclear norm, our TGLRR method can obtain a better performance by the TNN, and solve the non-convex and discontinuous issues. Secondly, the TGLRR method can seize the valuable information lying in data manifold and feature manifold simultaneously. Finally, the TGLRR method can capture the internal similarity information and some underlying affinity among data points by incorporating a graph regularization term and utilizing a linear association of some bases to represent each data point.

The remainder of this article is organized as follows. In the Results section, TGLRR is exploited for clustering and feature selection on integrated gene expression data. In Conclusions section, conclusions and the future work are given. In Methods section, our TGLRR method is put forward and the optimization problem is resolved through an efficient framework based on LADMAP [27].

## Results

### Integrative gene expression datasets

To validate the performance of TGLRR model, six clustering experiments and a feature selection experiment are conducted. The experimental data are integrative cancer gene expression data instead of single cancer data for avoiding sample imbalance problem. Seven different datasets are produced via integrating five different single gene expression data downloaded from The Cancer Genome Atlas (TCGA, https://www.cancer.gov/about-nci/organization/ccg/research/structural-genomics/tcga). The pertinent information about the seven integrative datasets is listed in Table 1.

**Table 1:** Description about seven integrative gene expression datasets

Datasets	Genes	Samples	Samples classes
PAAD-COAD	20502	176-262	2
HNSC-ESCA	20502	398-183	2
CHOL-HNSC-ESCA	20502	36-398-183	3
COAD-PAAD-ESCA	20502	262-176-183	3
PAAD-ESCA-HNSC	20502	180-192-418	3
HNSC-PAAD-CHOL-ESCA	20502	398-176-36-183	4
ESCA-COAD-CHOL-PAAD	20502	183-262-36-176	4

PAAD, ESCA, COAD, CHOL and HNSC are the abbreviations of Pancreatic Ductal Adenocarcinoma, Esophageal Carcinoma, Colorectal Adenocarcinoma, Cholangiocarcinoma, Head and Neck Squamous Cell Carcinoma, respectively. Taking PAAD-COAD dataset for example, it is only composed of the tumor samples of PAAD and COAD data, in which PAAD data are made up of 176 tumor samples and 4 normal samples, and COAD data consist of 262 tumor samples and 19 normal samples. HNSC-ESCA, CHOL-HNSC-ESCA, COAD-PAAD-ESCA, HNSC-PAAD-CHOL-ESCA and ESCA-COAD-CHOL-PAAD datasets are also made in the production way of PAAD-COAD dataset. But PAAD-ESCA-HNSC dataset is composed of the whole samples of PAAD, ESCA and HNSC.

To eliminate redundant features and avoid over-fitting, the dimension of data matrix **X** is reduced before clustering and feature selection experiment, which can also greatly abate the computational cost. PCA is chosen for dimension reduction experiments in our paper. In addition, 2000-dimensional data **X** are obtained after dimension reduction.

### Parameters selection

**Fig. 1:**
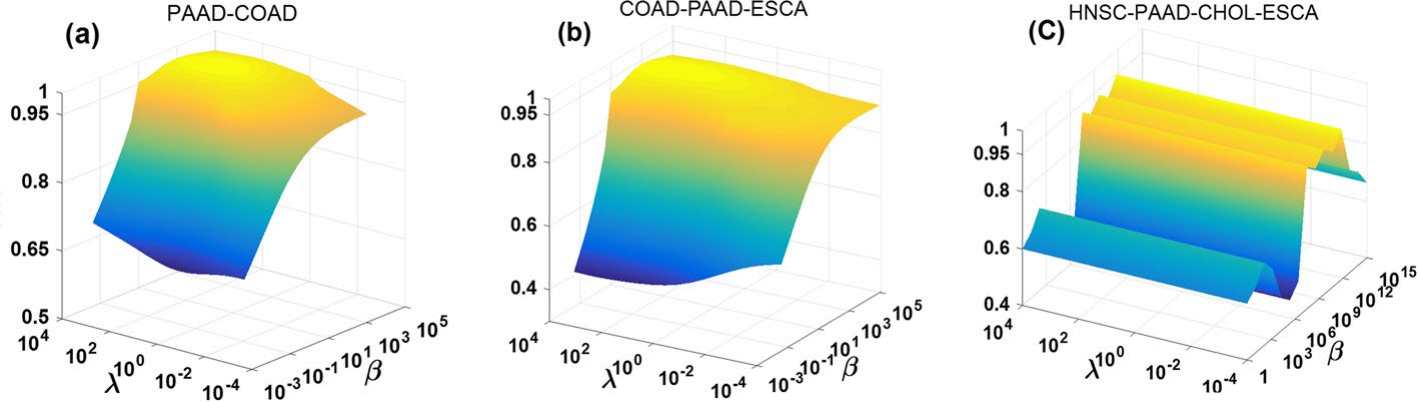
The clustering performance of TGLRR model versus parameter $\lambda$ and $\beta$. **a** the clustering results on PAAD-COAD dataset, **b** the results on COAD-PAAD-ESCA dataset, **c** the results on HNSC-PAAD-CHOL-ESCA dataset

There are three important parameters, *i.e.* regularization terms $\lambda$, $\beta$ and *r* of the TNN in our TGLRR model. The grid search is used to pick up the values of $\lambda$, $\beta$ and *r*. Figure 1 shows that clustering results are varied with the parameters $\lambda$ and $\beta$ on three distinct integrative datasets of tumor gene expression.

X-axis represents the values range of $\lambda$, Y-axis represents the values range of $\beta$, and Z-axis represents the clustering accuracy in Fig. 1. It can be distinctly found that the effect of clustering accuracy stems from $\beta$ that is greater than the effect from $\lambda$, especially in HNSC-PAAD-CHOL-ESCA dataset. Finally, it can be found that TGLRR performs well when $\lambda = 10$ and $\beta = 10^{4}$ on PAAD-COAD dataset, $\lambda = 10^{ - 2}$ and $\beta = 10$ on HNSC-ESCA dataset, $\lambda = 10^{ - 2}$ and $\beta = 10^{2}$ on CHOL-HNSC-ESCA dataset, $\lambda = 10^{ - 1}$ and $\beta = 10^{4}$ on COAD-PAAD-ESCA dataset, $\lambda = 10^{ - 2}$ and $\beta = 10^{11}$ on HNSC-PAAD-CHOL-ESCA dataset, and $\lambda = 10^{2}$ and $\beta = 10^{10}$ on ESCA-COAD-CHOL-PAAD dataset, respectively.

**Fig. 2:**
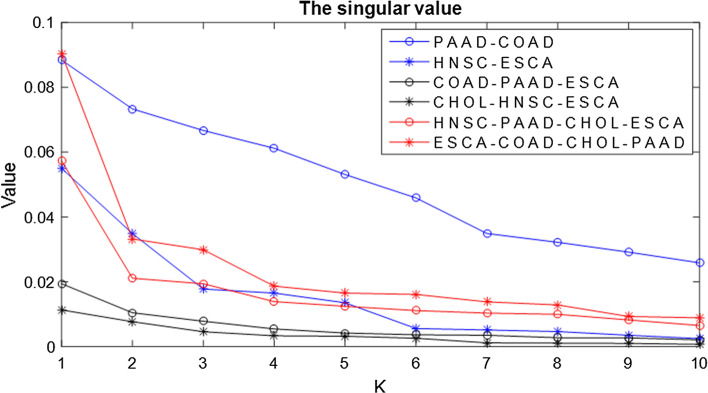
The singular values on six distinct matrices

Different from the method in [25] that tries all the possible values to seek the optimal value of *r*, the method in [28] is used to choose the optimal value of *r*. A curve graph showing the singular values needs to be drawn. Figure 2 shows the summary curve graph on six datasets applied in clustering experiments.

X-axis indicates the number and Y-axis denotes the singular values in Fig. 2. The value of the first inflection point in each curve is chosen as the value of *r* corresponding to each dataset. The principle of selecting *r* is that the singular values before inflection point are bigger than the singular values after inflection point. Therefore, the values of *r* on PAAD-COAD, HNSC-ESCA, CHOL-HNSC-ESCA, COAD-PAAD-ESCA, HNSC-PAAD-CHOL-ESCA and ESCA-COAD-CHOL-PAAD datasets are set as 2, 3, 3, 2, 2 and 3, respectively.

### Convergence analysis

**Fig. 3:**
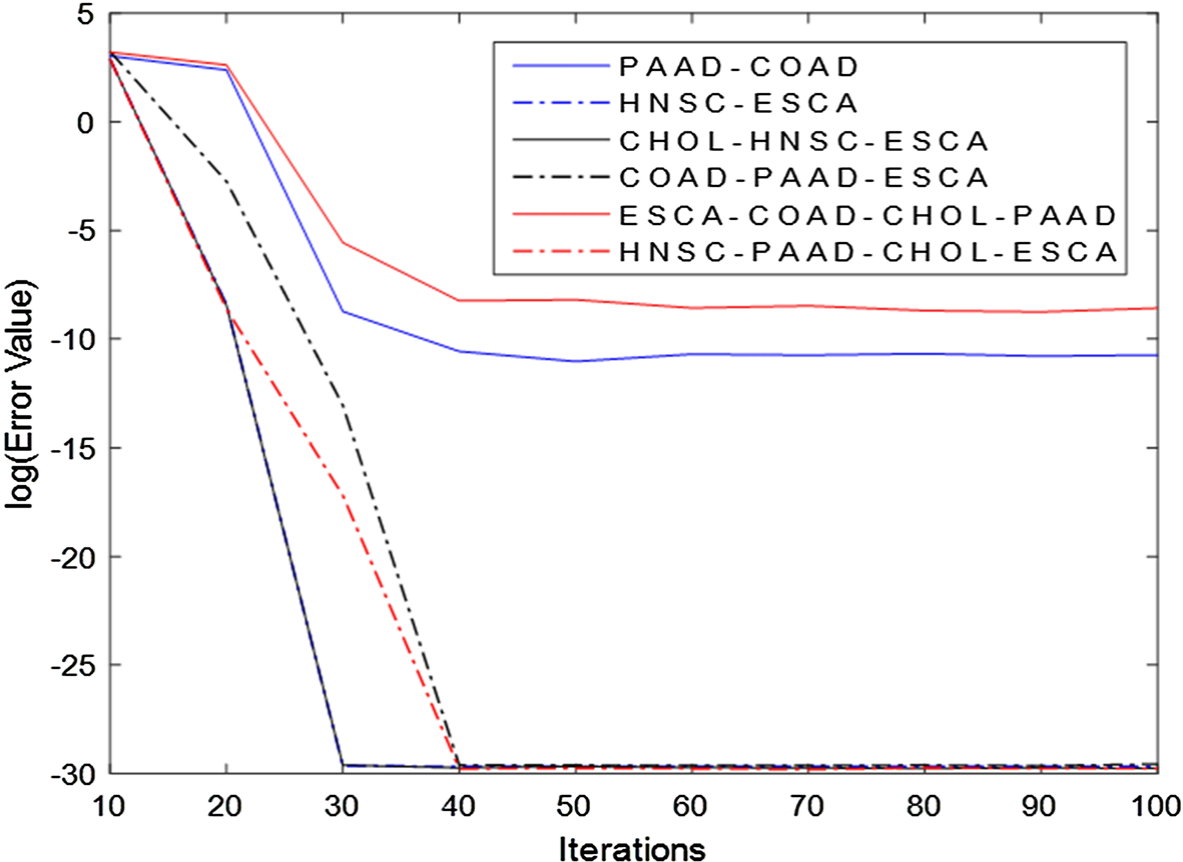
Convergence curves of TGLRR on gene expression data

Since Algorithm 1 is a practical application based on LADMAP framework whose convergence has been proved in [27], Algorithm 1 should also be convergent. Many approaches are able to demonstrate the convergence property of algorithms [23, 29]. In our paper, an efficient approach in [29] by means of auxiliary function is exploited to validate the convergence property of TGLRR method. The results are exhibited in Fig. 3.

The abscissa in Fig. 3 indicates the iteration number and the ordinate denotes the loss function value. As shown in Fig. 3, our model is convergent. The TGLRR method begins to converge after 30 iterations on two datasets, such as HNSC-ESCA and CHOL-HNSC-ESCA. On other four datasets, the TGLRR method converges in 40 iterations. Here, the HNSC-ESCA dataset may be easily addressed, so our method begins to converge on the two datasets after 30 iterations while it needs 40 iterations on the other four datasets.

### Clustering results

In this subsection, the TGLRR is applied for clustering, and compared with K-means, LLRR [14], LRR [30], RPCA [5], DGLRR [31], and LatLRR [22].

In respect of the dictionary matrix **X**, the optimal solution ${\mathbf{Z}}^{*}$ to TGLRR is able to symbolize “the minimum rank representation” of the data matrix **X**. What’s more, the *i*-th column about **Z** could be regarded as a “better” reflection of the *i*-th column about **X** so as to make the subspace structure more easily detectable [31]. Namely, the optimal solution ${\mathbf{Z}}^{*}$ could include almost all the sample information about integrative gene expression data **X**. Therefore, ${\mathbf{Z}}^{*}$ can be used for clustering experiments by K-means.

To measure the performance of our approach, three quantity metrics are adopted in this paper, *i.e.*, accuracy (ACC), normalized mutual information (NMI) and F-measure. As a widely used metric in machine learning field, ACC can be defined as follows:

1 $${{\text{ACC}} }=\frac{{\sum\nolimits_{i = 1}^{n} {\updelta \left( {\mathop {\text{L}}\limits^{ \wedge } \left( i \right), {\text{Map}}\left( {l_{i} } \right)} \right)} }}{n},$$

where *n* indicates the total number of tumor samples in an integrated data, $\updelta \left( {x,y} \right)$ is a delta function set to 1 only when $x = y$ and 0 otherwise, $\hat{L}\left( i \right)$ denotes true class label of the *i*-th sample, and $l_{i}$ represents the cluster label produced by the algorithms. ${\text{Map}}\left( {l_{i} } \right)$ is a mapping function permuting every $l_{i}$ to match real sample label.

The second index of NMI is defined by

2 $${\text{NMI}}\left( {{\text{T}},{\hat{{\text{T}}}}} \right) = {{{{\text{MI}}}\left( {{{\text{T}}},{\hat{{\text{T}}}}} \right)} \mathord{\left/ {\vphantom {{{{\text{MI}}}\left( {{\text{T}},{\hat{{\text{T}}}}} \right)} {\max \left( {{{\text{H}}}\left( {{\text{T}}} \right),{\text{H}}\left( {{\hat{{\text{T}}}}} \right)} \right)}}} \right. \kern-\nulldelimiterspace} {\max \left( {{{\text{H}}}\left( {{\text{T}}} \right),{{\text{H}}}\left( {{\hat{{\text{T}}}}} \right)} \right)}},$$

where T and $\hat{T}$ denote two different tumor index sets separately. H(T) and ${{\text{H}}}\left( {{\hat{{\text{T}}}}} \right)$ represent the entropy in T and $\hat{T}$, respectively. And ${{\text{MI}}}\left( {{{\text{T}}},\hat{T}} \right) = \sum\limits_{{t \in {{\text{T}}}}} {\sum\limits_{{\hat{t} \in \hat{T}}} {{{\text{P}}}\left( {t,\hat{t}} \right)} } \log {{{{\text{P}}}\left( {t,\hat{t}} \right)} \mathord{\left/ {\vphantom {{{{\text{P}}}\left( {t,\hat{t}} \right)} {\left( {{{\text{P}}}\left( t \right){{\text{P}}}\left( {\hat{t}} \right)} \right)}}} \right. \kern-\nulldelimiterspace} {\left( {{{\text{P}}}\left( t \right){{\text{P}}}\left( {\hat{t}} \right)} \right)}}$ where P(*t*) is the marginal probability distribution function, namely, the probabilities that a tumor sample arbitrarily chosen from an integrated dataset belongs to cluster T. In addition, ${{\text{MI}}}\left( {{\text{T}},\hat{{\text{T}}}} \right)$ indicates the joint probabilities that a tumor sample belongs to the two clusters T and ${\hat{{\text{T}}}}$ simultaneously.

F-measure is the comprehensive evaluation index considering both precision and recall, and written as:

3 $${{\text{F}}} - {{\text{measeure}}}=2 \cdot {{\left( {{\text{recall}} \cdot {{\text{precision}}}} \right)} \mathord{\left/ {\vphantom {{\left( {{\text{recall}} \cdot {{\text{precision}}}} \right)} {\left( {{\text{recall}} + {\text{precision}}} \right)}}} \right. \kern-\nulldelimiterspace} {\left( {{\text{recall}} + {{\text{precision}}}} \right)}},$$

where ${\text{recall = }}{{{{\text{TP}}}} \mathord{\left/ {\vphantom {{{{\text{TP}}}} {\left( {{{\text{TP}}} + {{\text{FN}}}} \right)}}} \right. \kern-\nulldelimiterspace} {\left( {{{\text{TP}}} + {{\text{FN}}}} \right)}}$ and ${\text{precision}} ={{{{\text{TP}}}} \mathord{\left/ {\vphantom {{{{\text{TP}}}} {\left( {{{\text{TP}}} + {{\text{FP}}}} \right)}}} \right. \kern-\nulldelimiterspace} {\left( {{{\text{TP}}} + {{\text{FP}}}} \right)}}$. TP, FP, TN and FN indicate the true-positive, false-positive, true-negative and false-negative, respectively.

To prove the effectiveness of TGLRR, the detailed clustering results of these methods on integrative tumor gene expression data are listed by three tables. In tables, the values about ACC, NMI and F-measure are the average of 100 clustering results of each approach, and the values on the right of ± are the variance of 100 results.

Table 2 reports the clustering results on PAAD-COAD and HNSC-ESCA datasets. Obviously, our TGLRR method exceeds other six comparison methods on PAAD-COAD dataset. TGLRR is more robust than other six methods on PAAD-COAD dataset from the point of the variance values. HNSC-ESCA data are extraordinary and may be easily addressed, in which the clustering results about all algorithms are good and particularly the evaluation indices of RPCA and TGLRR are 1.

**Table 2:** The clustering results on PAAD-COAD and HNSC-ESCA integrative data

	PAAD-COAD			HNSC-ESCA		
	ACC(%)	NMI(%)	F-measure(%)	ACC(%)	NMI(%)	F-measure(%)
K-means	91.57 ± 0.89	68.77 ± 4.24	91.62 ± 1.01	99.36 ± 0.05	98.00 ± 0.50	98.81 ± 0.18
LLRR	93.95 ± 0.29	71.59 ± 1.29	93.83 ± 0.26	99.83 ± 0.00	98.07 ± 0.00	99.80 ± 0.00
LRR	93.63 ± 0.57	70.70 ± 2.52	93.64 ± 0.51	99.83 ± 0.00	98.07 ± 0.00	99.80 ± 0.00
RPCA	93.81 ± 0.46	71.09 ± 2.27	93.81 ± 0.42	100.00 ± 0.00	100.00 ± 0.00	100.00 ± 0.00
DGLRR	94.14 ± 0.40	71.98 ± 1.80	94.13 ± 0.47	99.83 ± 0.00	98.07 ± 0.00	99.80 ± 0.00
LatLRR	93.76 ± 0.33	71.46 ± 1.50	93.77 ± 0.29	99.83 ± 0.00	98.07 ± 0.00	99.80 ± 0.00
TGLRR	95.15 ± 0.00	74.44 ± 0.00	95.10 ± 0.00	100.00 ± 0.00	100.00 ± 0.00	100.00 ± 0.00

The clustering results on two integrated datasets containing three types of tumors are exhibited in Table 3. It can be seen that the clustering performance of TGLRR model outperforms other models on COAD-PAAD-ESCA dataset. On CHOL-HNSC-ESCA dataset, TGLRR’s ACC, NMI and F-measure values are higher than values obtained via other five models except for LRR. Consequently, it still can be said that the TGLRR method outstrips other methods on CHOL-HNSC-ESCA dataset.

**Table 3:** The clustering results on CHOL-HNSC-ESCA and COAD-PAAD-ESCA data

	CHOL-HNSC-ESCA			COAD-PAAD-ESCA		
	ACC (%)	NMI (%)	F-measure (%)	ACC (%)	NMI (%)	F-measure (%)
K-means	83.49 ± 1.77	76.23 ± 3.47	77.05 ± 3.42	83.61 ± 3.25	76.11 ± 2.85	81.95 ± 4.28
LLRR	96.73 ± 0.80	94.80 ± 1.26	94.69 ± 2.09	87.07 ± 2.23	79.50 ± 1.46	86.19 ± 2.94
LRR	97.13 ± 0.42	95.32 ± 0.61	96.16 ± 0.82	88.16 ± 1.98	80.27 ± 1.48	87.47 ± 2.53
RPCA	85.40 ± 2.64	81.43 ± 4.16	81.26 ± 4.59	85.59 ± 2.72	78.85 ± 2.39	83.98 ± 3.49
DGLRR	94.70 ± 1.03	92.33 ± 1.78	91.63 ± 2.62	86.14 ± 2.38	78.67 ± 1.82	84.93 ± 3.11
LatLRR	93.94 ± 1.57	91.37 ± 2.46	91.57 ± 3.32	87.16 ± 2.52	79.33 ± 1.93	86.16 ± 3.30
TGLRR	98.37 ± 0.00	90.58 ± 0.03	96.09 ± 0.01	92.82 ± 0.77	79.51 ± 0.93	92.62 ± 0.91

From Table 4, our TGLRR method outmatches other six methods on HNSC-PAAD-CHOL-ESCA and ESCA-COAD-CHOL-PAAD datasets.

**Table 4:** The clustering results on HNSC-PAAD-CHOL-ESCA and ESCA-COAD-CHOL-PAAD data

	HNSC-PAAD-CHOL-ESCA			ESCA-COAD-CHOL-PAAD		
	ACC (%)	NMI (%)	F-measure (%)	ACC (%)	NMI (%)	F-measure (%)
K-means	78.42 ± 0.94	71.34 ± 1.03	72.19 ± 1.92	82.49 ± 2.15	77.01 ± 1.76	75.71 ± 3.30
LLRR	87.66 ± 0.94	75.56 ± 0.40	86.90 ± 2.04	84.41 ± 2.07	80.24 ± 1.22	82.60 ± 2.73
LRR	88.63 ± 0.39	75.89 ± 0.21	89.16 ± 0.81	87.40 ± 2.05	82.52 ± 1.20	87.62 ± 2.17
RPCA	84.85 ± 1.54	80.29 ± 1.50	81.72 ± 2.57	83.39 ± 1.73	79.28 ± 1.31	76.86 ± 3.10
DGLRR	86.68 ± 0.85	75.22 ± 0.41	84.99 ± 1.94	85.99 ± 2.26	81.52 ± 1.39	84.01 ± 3.13
LatLRR	85.14 ± 1.02	73.96 ± 0.43	84.26 ± 2.21	86.04 ± 1.85	81.49 ± 1.14	82.37 ± 1.94
TGLRR	93.46 ± 0.93	82.83 ± 0.75	90.90 ± 1.20	90.62 ± 1.53	79.87 ± 1.49	90.34 ± 1.64

### Feature selection

Cancers are commonly relevant to gene mutation or abnormal expression of genes. Thus, in this subsection, the TGLRR method is used to identify co-feature genes of PAAD, ESCA and HNSC from PAAD-ESCA-HNSC dataset.

From the formula (14), a minimum solution ${\mathbf{G}}^{*}$ can be got from an integrative gene expression data **X** via TGLRR scheme. ${\mathbf{G}}^{*}$ can obtain the feature manifold structure lying in data. As a result, it can be applied in feature gene extraction. From the view of cancer, its pathogenesis may be related to gene mutation [32]. It is extremely meaningful to find out the feature genes inducing cancers from gene expression data.

Similar to the subsection of Parameters Selection, 10^−2^, 10^3^ and 4 are assigned to $\lambda$, $\beta$ and *r*.

Table 5 exhibits the top 10 co-feature genes with the mean of highest relevance score distinguished by the TGLRR method from PAAD-ESCA-HNSC dataset. The related diseases, related pathways and coded proteins about these genes are gotten from GeneCards (https://www.genecards.org/). These genes are most likely to lead to PAAD, ESCA and HNSC simultaneously.

**Table 5:** The top 10 genes selected via TGLRR on PAAD-ESCA-HNSC

Gene ED	Relevance score	Related diseases	Coded proteins
CDH1	101.03, 96.95, 124.3, 107.43	Gastric, breast, colorectal, thyroid and ovarian cancer	Cadherin superfamily
TGFB1	73.21, 44.14, 76.66, 64.67	Camurati-Engelmann disease, Encephalopathy, Inflammatory Bowel Disease and Immunodeficiency	Transforming Growth Factor-Beta Superfamily of Proteins
RELA	27.63, 11.33, 41.36, 26.77	Mucocutaneous Ulceration, Chronic and Ependymoma	Transcription Factor
ANXA5	26.80, 10.31, 42.30, 26.47	Pregnancy Loss, Recurrent 3 and Antiphospholipid Syndrome	Calcium-Dependent Phospholipid Binding Proteins
RHOA	27.48, 11.81, 31.46, 23.58	Adenocarcinoma and Peripheral T-Cell Lymphoma	Rho Family of Small GTPases
PTPN11	13.04, 13.56, 43.23, 23.28	Noonan Syndrome 1 and Juvenile Myelomonocytic Leukemia	Protein Tyrosine Phosphatase
CTNNA1	20.94, 19.40, 24.80, 21.71	Macular Dystrophy, Patterned, 2 and Butterfly-Shaped Pigment Dystrophy	Cell Adhesion Process Protein
IGF2R	13.40, 19.07, 25.26, 19.24	Hepatocellular Carcinoma and Inclusion-Cell Disease	Receptor for Both Insulin-Like Growth Factor 2 and Mannose 6-Phosphate
RUNX1	10.85, 12.97, 25.61, 16.48	Platelet Disorder, Familial, with Associated Myeloid Malignancy, leukemia and Isolated Delta-Storage Pool Disease	Transcription Factor
EWSR1	12.55, 9.19, 27.33, 16.36	Ewing Sarcoma and Desmoplastic Small Round Cell Tumor	Multifunctional Protein

Take the contents in the second column of the second row as an example, the first, second and third numeral are the relevance score of CDH1 gene to PAAD, ESCA and HNSC, respectively, and the fourth is the mean

From Table 5, clearly, CDH1 gene with the highest relevance score can result in a host of cancers, which indicates that CDH1 may be a dangerous co-feature gene. What's more, PAAD, ESCA and HNSC are all correlative with CDH1 and RHOA, which can be affirmed from [33–38]. It is a verifiable fact that TGFB1 and RELA all serve as a predictor for PAAD and ESCA via consulting some literatures. Some data show that PTPN11 may induce HNSC and PAAD. From [39, 40], it can be seen that ESCA is relevant to IGF2R and RUNX1. In addition, the related pathways of RUNX1 and EWSR1 include transcriptional misregulation in cancer. So, RUNX1 and EWSR1 may be co-characteristic genes of PAAD, ESCA and HNSC.

All in all, the TGLRR method is successful in identifying co-characteristic genes on the integrative gene expression datasets.

## Discussions

The TGLRR method is applied to the tumor clustering and gene selection, and superior to the other methods. Based on above results, it can be affirmed that the TNN could capture more valuable information existed in data than the nuclear norm from data. By comparing the results of DGLRR, a conclusion can be drawn that the graph Laplacian regularization imposed on feature manifold may cause adverse effects for clustering on our integrative datasets. The TGLRR method has some limitations. For example, on HNSC-PAAD-CHOL-ESCA dataset, the variance values of TGLRR is larger than LRR. It may be caused by the integrated datasets and its stability needs to be improved in future.

In a word, these improvements to the prevenient LRR model can help TGLRR catch more useful information concealed in the low-dimensional manifold structure.

## Conclusions

The paper proposes a Low-Rank Representation approach called TGLRR. It can capture the global and local geometric structures in data manifold via using the raw data matrix as the dictionary matrix and introducing the graph-Laplacian regularization term. Furthermore, TGLRR can gain a better approximation to the rank operator than the approaches regularized by the nuclear norm. The objective function of TGLRR is perfectly resolved through an iterative algorithm based on LADMAP framework. The efficiency and robustness of our TGLRR method are testified through the encouraging experimental results.

## Methods

### Related LRR methods

Based on the assumption that the observation data **X** are sampled from a union of several low-dimensional subspaces $S = \sum {S_{1} ,S_{2} , \ldots ,S_{k} }$ located in a high-dimensional spaces, LRR was raised in [16]. If data are noiseless, the rank minimization problem of LRR is written into

4 $$\mathop {\min }\limits_{{\mathbf{Z}}} {{\text{rank}}}\left( {\mathbf{Z}} \right), \, s. \, t. \, {\mathbf{X}} = {\mathbf{DZ}},$$

where ${\mathbf{X}} = \left[ {{\mathbf{x}}_{1} ,{\mathbf{x}}_{2} , \cdots ,{\mathbf{x}}_{n} } \right] \in {\mathbf{R}}^{m \times n}$ is the original data matrix and ${\mathbf{Z}} \in {\mathbf{R}}^{n \times n}$ is a low-rank matrix recovered from **X** via LRR. ${\mathbf{D}} \in {\mathbf{R}}^{m \times n}$ is a basis matrix (or named dictionary matrix), which spans the whole data space linearly. The observation data generally exist more or less noise in real life, so the optimization problem (4) may be impracticable. The LRR model with noise is

5 $$\mathop {\min }\limits_{{{\mathbf{Z}},{\mathbf{P}}}} {{\text{rank}}}\left( {\mathbf{Z}} \right) + \lambda \left\| {\mathbf{P}} \right\|_{0} , \, s. \, t. \, {\mathbf{X}} = {\mathbf{DZ}} + {\mathbf{P}},$$

where ${\mathbf{P}} \in {\mathbf{R}}^{m \times n}$ is the reconstruction errors matrix (or called noise matrix). $\lambda$ is a penalty parameter aiming to adjust the sparsity of matrix **P** and the reconstruction fidelity of data matrix **X** damaged by errors matrix **P**. $\left\| {\mathbf{P}} \right\|_{0}$ is the L_0_-norm of matrix **P**, which indicates the number of non-zero elements in matrix **P**.

Since the rank function is discrete, the problem (5) may have multiple solutions and the *L*_0_-minimization is non-convexity and intractable. Usually, solving the problem (5) is NP-hard [41]. To better solve the above rank minimization problem, the nuclear norm is imposed on the low-rank matrix, and the *L*_0_-norm is replaced with the *L*_2,1_-norm [17]. The convex optimization problem about LRR model is written as follows:

6 $$\mathop {\min }\limits_{{{\mathbf{Z}},{\mathbf{P}}}} \left\| {\mathbf{Z}} \right\|_{*} + \lambda \left\| {\mathbf{P}} \right\|_{2,1} , \, s. \, t. \, {\mathbf{X}} = {\mathbf{DZ}} + {\mathbf{P}},$$

where $\left\| {\mathbf{Z}} \right\|_{*} = \sum\nolimits_{i}^{{\min \left( {m,n} \right)}} {\sigma_{i} \left( {\mathbf{Z}} \right)}$($\updelta _{i} \left( {\mathbf{Z}} \right)$ is the *i*-th largest singular value of **Z**) denotes the nuclear norm of matrix **Z**, and $\left\| {\mathbf{P}} \right\|_{{2,1}} = \sum\nolimits_{i = 1}^{m} {\left( {\sum\nolimits_{j = 1}^{n} {{{\text{m}}}_{ij}^{{2}} } } \right)}^{{{{1} \mathord{\left/ {\vphantom {{1} {2}}} \right. \kern-\nulldelimiterspace} {2}}}}$ denotes the *L*_2,1_-norm of matrix **P**. To get a self-expression model, the observation data **X** are generally installed as the dictionary matrix [13, 14, 22]. The final LRR model becomes

7 $$\mathop {\min }\limits_{{{\mathbf{Z}},{\mathbf{P}}}} \left\| {\mathbf{Z}} \right\|_{*} + \lambda \left\| {\mathbf{P}} \right\|_{2,1} , \, s. \, t. \, {\mathbf{X}} = {\mathbf{XZ}} + {\mathbf{P}}.$$

For low-rank matrix ${\mathbf{Z}} = \left[ {{\mathbf{z}}_{1} ,{\mathbf{z}}_{2} , \cdots ,{\mathbf{z}}_{n} } \right]$, its each element ${\mathbf{z}}_{ij}$ can reflect the manifold information, *i.e.* the similarity between the data point ${\mathbf{x}}_{i}$ and the data point ${\mathbf{x}}_{j}$. Therefore, matrix **Z** can be seen as an affinity matrix [14]. LRR is devoted to seek the lowest rank representation of the observation data. With the help of an appropriate dictionary matrix, the underlying row space can be recovered via the lowest rank representation such that the true segmentation of data can be correctly revealed. Thus, LRR method can manage the data extracted from a union of multiple subspaces well [17].

Nevertheless, LRR method has to face two issues owing to the raw data **X** that are used as the basis. First, LRR method requires that the basis contains adequate data samples from the subspaces so as to possess the capacity of representing the underlying subspaces. Second, LRR method demands that noise of data **X** is little, *i.e.* only a part of **X** is corrupted. To remedy these two shortcomings of LRR, Liu et al*.* proposed the following convex optimization LRR problem [22]:

8 $$\mathop {\min }\limits_{{{\mathbf{Z}},{\mathbf{G}},{\mathbf{P}}}} \left\| {\mathbf{Z}} \right\|_{*} + \left\| {\mathbf{G}} \right\|_{*} + \lambda \left\| {\mathbf{P}} \right\|_{1} , \, s. \, t. \, {\mathbf{X}} = {\mathbf{XZ}} + {\mathbf{GX}} + {\mathbf{P}},$$

where $\left\| {\mathbf{P}} \right\|_{1} = \sum\nolimits_{i = 1}^{n} {\sum\nolimits_{j = 1}^{m} {\left| {{{\text{p}}}_{ij} } \right|} }$ is the *L*_1_-norm of matrix **P** and **G** is the feature matrix separated from the original **X**. Equation (8) is a state-of-the-art LRR-based subspace learning model, named LatLRR. By means of LatLRR model, the observed sampling can be expressed via many unobserved sampling effectively [42]. In practical application, **Z** and **G** are applied in cluster analysis and feature selection, respectively.

### Truncated nuclear norm (TNN)

The TNN is the summation of a few smaller singular values, *i.e.* the sum of some largest singular values is subtracted from the nuclear norm [24]. As an approximation of a rank operator, the largest *r*-th singular values could produce minor amount of information, meanwhile, the minimal $\left( {\min \left( {m,n} \right) - r} \right)$-th singular values act a crucial role [23]. Compared to the nuclear norm, the TNN may be a better approximation to the rank operator. Its mathematical formula is

9 $$\left\| {\mathbf{Z}} \right\|_{r} = \sum\nolimits_{i = r + 1}^{{\min \left( {m,n} \right)}}\updelta _{i} \left( {\mathbf{Z}} \right),$$

where $\updelta _{i} \left( {\mathbf{Z}} \right)$ denotes the *i*-th largest singular value belongs to **Z** and r is a nonnegative integer and ${\text{r}} \le min\left( {m, \, n} \right)$.

Since the minimization of Eq. (9) is not convex, it cannot be directly resolved through the approaches. For overcoming this issue, Hu et al*.* come up with a theorem [25]. According to the Theorem, the equivalent transformation of Eq. (9) is achieved.

10 $$\left\| {\mathbf{Z}} \right\|_{r} = \sum\nolimits_{i = r + 1}^{{\min \left( {m,n} \right)}}\updelta _{i} \left( {\mathbf{Z}} \right) = \left\| {\mathbf{Z}} \right\|_{*} - \mathop {{{\text{max}}}}\limits_{{{\mathbf{AA}}^{{\text{T}}} { = }{\mathbf{I}}, \, {\mathbf{BB}}^{T} = {\mathbf{I}}}} {{\text{Tr}}}\left( {{\mathbf{AZB}}^{T} } \right).$$

### Graph-Laplacian regularization

Graph-Laplacian regularization is an outstanding manifold learning method, which can uncover the internal geometrical structures among the data points. As a result, naturally, appears a number of LRR models regularized by graph embedding manifold regularization [13, 43].

Given a *k*-nearest-neighbor graph G, suppose it has *n* vertices, and each vertex denotes a data point hidden in an underlying sub-manifold M [11]. Then, a symmetric weight matrix ${\mathbf{W}} \in {\mathbf{R}}^{n \times n}$ is constructed, where ${\mathbf{w}}_{ij}$ expresses the *i* weight of the edge linking vertices *i* and *j*. The value of every ${\mathbf{w}}_{ij}$ can be calculated via

11 $${\mathbf{w}}_{ij} { = }\left\{ {\begin{array}{*{20}c} {1,} & {{{\text{if}}}\;{\mathbf{y}}_{i} \in {{\text{N}}}_{k} \left( {{\mathbf{d}}_{j} } \right)\;{\text{or}}\;{\mathbf{y}}_{j} \in {{\text{N}}}_{k} \left( {{\mathbf{d}}_{i} } \right),} \\ {0,} & {{{\text{otherwise}}},} \\ \end{array} } \right.$$

where ${{\text{N}}}_{k} \left( {{\mathbf{d}}_{j} } \right)$ indicates the *k*-nearest-neighbors of data point ${\mathbf{d}}_{j}$.

Next, a diagonal matrix **O**, termed a degree matrix, need to be established. The value of the *i*-th member of **O** can be calculated by the sum of all the similarities associated with vertex ${\mathbf{d}}_{j}$, *i.e.* ${\mathbf{o}}_{ii} = \sum\nolimits_{j} {{\mathbf{w}}_{ij} }$. The graph-Laplacian matrix **L** can be obtained by

12 $${\mathbf{L}} = {\mathbf{O}} - {\mathbf{W}}.$$

Finally, the graph embedding regularization term can be formulated by

13 $${{\text{Tr}}}\left( {{\mathbf{ZLZ}}^{T} } \right).$$

### Truncated nuclear norm and graph-Laplacian regularized low-rank representation method

Motivated by strengthening the robustness of LRR, our method (TGLRR) is put forward. Considering that some data may exist nonlinear geometric structure [14] and the disadvantages about the nuclear norm, the TNN and graph embedding manifold learning are introduced into our rank minimization problem to extract more essential information hidden in data. The objective function of TGLRR is formulated as follows:

14 $$\begin{gathered} \min \left\| {\mathbf{Z}} \right\|_{*} - \mathop {{{\text{max}}}}\limits_{{{\mathbf{AA}}^{{\text{T}}} { = }{\mathbf{I}}, \, {\mathbf{BB}}^{T} = {\mathbf{I}}}} {{\text{Tr}}}\left( {{\mathbf{AZB}}^{T} } \right) + \left\| {\mathbf{G}} \right\|_{*} {\kern 1pt} + \frac{\beta }{2}{{\text{Tr}}}\left( {{\mathbf{ZLZ}}^{T} } \right) + \lambda \left\| {\mathbf{P}} \right\|_{1} , \\ s. \, t. \, {\mathbf{X}} = {\mathbf{XZ}} + {\mathbf{GX}} + {\mathbf{P}}, \\ \end{gathered}$$

where $\beta \ge 0$ and $\lambda \ge 0$ are the regularization parameters for balancing the contribution of each term.

Essentially, TGLRR can get a more precise approximation to the rank function with the help of the TNN than the nuclear norm. And the underlying low-dimensional structures of data could be captured by the aid of the graph-Laplacian regularization and the basis matrix **X**.

### Optimization solution

To correctly solve the optimization problem of (14), an efficient iterative algorithm based on LADMAP framework is designed. The algorithm (Algorithm 1) is implemented via alternating two iterative procedures till Eq. (14) converges to the minimum.

The first step is to determine matrix **A** and **B**.

**Step 1**: Given ${\mathbf{Z}}_{k}$ (*k* indicates the *k*-th updating), the SVD (Singular Value Decomposition) of ${\mathbf{Z}}_{k}$ need to be conducted. $\left[ {{\mathbf{U}}_{k} ,{{\varvec{\Sigma}}}_{k} ,{\mathbf{V}}_{k} } \right] = {{\text{SVD}}}\left( {{\mathbf{Z}}_{k} } \right)$, where ${\mathbf{U}}_{k} = \left( {{\mathbf{u}}_{1} ,{\mathbf{u}}_{2} , \ldots ,{\mathbf{u}}_{m} } \right) \in {\mathbf{R}}^{m \times m}$, ${{\varvec{\Sigma}}}_{k} \in {\mathbf{R}}^{m \times n}$ and ${\mathbf{V}}_{k} = \left( {{\mathbf{v}}_{1} ,{\mathbf{v}}_{2} , \ldots ,{\mathbf{v}}_{m} } \right) \in {\mathbf{R}}^{n \times n}$. ${\mathbf{A}}_{k}$ and ${\mathbf{B}}_{k}$ are calculated via ${\mathbf{A}}_{k} = \left( {{\mathbf{u}}_{{1}} ,{\mathbf{u}}_{{2}} , \ldots ,{\mathbf{u}}_{r} } \right)^{T}$ and ${\mathbf{B}}_{k} = \left( {{\mathbf{v}}_{{1}} ,{\mathbf{v}}_{{2}} , \ldots ,{\mathbf{v}}_{r} } \right)^{T}$.

The second step is to resolve the following convex optimization problem:

15 $$\begin{gathered} \left[ {{\mathbf{Z}},{\mathbf{G}},{\mathbf{P}}} \right] = \arg \mathop {\min }\limits_{{{\mathbf{Z}},{\mathbf{G}},{\mathbf{P}}}} \left\| {\mathbf{Z}} \right\|_{*} - {{\text{Tr}}}\left( {{\mathbf{AZB}}^{T} } \right) + \left\| {\mathbf{G}} \right\|_{*} {\kern 1pt} + \frac{\beta }{2}{{\text{Tr}}}\left( {{\mathbf{ZLZ}}^{T} } \right) + \lambda \left\| {\mathbf{P}} \right\|_{1} , \\ s. \, t. \, {\mathbf{X}} = {\mathbf{XZ}} + {\mathbf{GX}} + {\mathbf{P}}. \\ \end{gathered}$$

**Step 2**: To achieve the separation of objective function (15), an auxiliary variable ${\mathbf{F}}$ is introduced. Equation (15) is rewritten as follows:

16 $$\begin{gathered} \min \left\| {\mathbf{Z}} \right\|_{*} - \mathop {{{\text{max}}}}\limits_{{{\mathbf{AA}}^{{\text{T}}} { = }{\mathbf{I}}, \, {\mathbf{BB}}^{T} = {\mathbf{I}}}} {{\text{Tr}}}\left( {{\mathbf{AFB}}^{T} } \right) + \left\| {\mathbf{G}} \right\|_{*} + \frac{\beta }{2}{{\text{Tr}}}\left( {{\mathbf{ZLZ}}^{T} } \right){\kern 1pt} + \lambda \left\| {\mathbf{P}} \right\|_{1} , \\ s. \, t. \, {\mathbf{X}} = {\mathbf{XZ}} + {\mathbf{GX}} + {\mathbf{P}},{\mathbf{Z}} = {\mathbf{F}}. \\ \end{gathered}$$

Equation (16) can be solved through LADMAP method, which introduces two Lagrangian multipliers **Y**^1^ and **Y**^2^. Thus, the augmented Lagrangian function can be defined as

17 $$\begin{aligned} & L\left( {{\mathbf{Z}}, \, {\mathbf{G}}, \, {\mathbf{F}}, \, {\mathbf{P}}, \,\upmu , \, {\mathbf{Y}}^{1} , \, {\mathbf{Y}}^{2} } \right) = \left\| {\mathbf{Z}} \right\|_{*} - {{\text{Tr}}}\left( {{\mathbf{AFB}}^{T} } \right) + \left\| {\mathbf{G}} \right\|_{*} \\ & \quad + \frac{\beta }{2}{{\text{Tr}}}\left( {{\mathbf{ZLZ}}^{T} } \right){\kern 1pt} + \lambda \left\| {\mathbf{P}} \right\|_{1} + \left\langle {{\mathbf{Y}}^{1} , \, {\mathbf{X}} - {\mathbf{XZ}} - {\mathbf{GX}} - {\mathbf{P}}} \right\rangle \\ & \quad + \left\langle {{\mathbf{Y}}^{2} , \, {\mathbf{Z}} - {\mathbf{F}}} \right\rangle {\kern 1pt} {\kern 1pt} {\kern 1pt} {\kern 1pt} {\kern 1pt} { + }{\upmu \mathord{\left/ {\vphantom {\upmu 2}} \right. \kern-\nulldelimiterspace} 2}\left\| {{\mathbf{X}} - {\mathbf{XZ}} - {\mathbf{GX}} - {\mathbf{P}}} \right\|_{F}^{2} + {\upmu \mathord{\left/ {\vphantom {\upmu 2}} \right. \kern-\nulldelimiterspace} 2}\left\| {{\mathbf{Z}} - {\mathbf{F}}} \right\|_{F}^{2} , \\ \end{aligned}$$

where $\upmu$ is the penalty parameter and $\left\| \cdot \right\|_{F}^{2}$ denotes the Frobenius norm of a matrix that is $\left\| {\mathbf{X}} \right\|_{F}^{2} = \sum\nolimits_{i = 1}^{m} {\sum\nolimits_{j = 1}^{n} {\left| {{\mathbf{x}}_{ij} } \right|^{2} } }$.

Next, the alternating minimization strategy is adopted to compute **Z**, **F**, **G** and **P**. In the iterative procedure, **Z**, **F**, **G** or **P** is updated when the other three variables are fixed, respectively.

**Updating Z**

To get the solution of **Z**, the below minimization objective *w.r.t.* **Z** needs to be solved.

18 $$\begin{aligned} {\mathbf{Z}}_{k + 1} & = \arg \mathop {\min }\limits_{{\mathbf{Z}}} L\left( {{\mathbf{Z}}, \, {\mathbf{G}}_{k} , \, {\mathbf{F}}_{k} , \, {\mathbf{P}}_{k} , \,\upmu _{k} , \, {\mathbf{Y}}_{{_{k} }}^{1} , \, {\mathbf{Y}}_{{_{k} }}^{2} } \right) \\ {\kern 1pt} {\kern 1pt} & = \arg \mathop {\min }\limits_{{\mathbf{Z}}} \left\| {\mathbf{Z}} \right\|_{*} + \frac{\beta }{2}{{\text{Tr}}}\left( {{\mathbf{ZLZ}}^{T} } \right){\kern 1pt} {\kern 1pt} + \left\langle {{\mathbf{Y}}_{k}^{1} , \, {\mathbf{X}} - {\mathbf{XZ}} - {\mathbf{G}}_{k} {\mathbf{X}} - {\mathbf{P}}_{k} } \right\rangle {\kern 1pt} \\ & \quad + \left\langle {{\mathbf{Y}}_{{_{k} }}^{2} , \, {\mathbf{Z}} - {\mathbf{F}}_{k} } \right\rangle { + }{{\upmu _{k} } \mathord{\left/ {\vphantom {{\upmu _{k} } 2}} \right. \kern-\nulldelimiterspace} 2}\left\| {{\mathbf{X}} - {\mathbf{XZ}} - {\mathbf{G}}_{k} {\mathbf{X}} - {\mathbf{P}}_{k} } \right\|_{F}^{2} + {{\upmu _{k} } \mathord{\left/ {\vphantom {{\upmu _{k} } 2}} \right. \kern-\nulldelimiterspace} 2}\left\| {{\mathbf{Z}} - {\mathbf{F}}_{k} } \right\|_{F}^{2} . \\ \end{aligned}$$

Equation (18) has a closed-form solution:

19 $${\mathbf{Z}}_{k + 1}^{*} = \Theta_{{{1 \mathord{\left/ {\vphantom {1 {\upeta _{1}\upmu _{k} }}} \right. \kern-\nulldelimiterspace} {\upeta _{1}\upmu _{k} }}}} \left( {{\mathbf{Z}}_{k} - {{\nabla_{zq} \left( {{\mathbf{Z}}_{k} } \right)} \mathord{\left/ {\vphantom {{\nabla_{zq} \left( {{\mathbf{Z}}_{k} } \right)} {\upeta _{1} }}} \right. \kern-\nulldelimiterspace} {\upeta _{1} }}} \right),$$

where $$\Theta \left( \cdot \right)$$ indicates the Singular Value Thresholding operator (SVT), $$\nabla_{zq} \left( {{\mathbf{Z}}_{k} } \right) = \frac{\beta }{2}\left( {{\mathbf{ZL}}^{T} + {\mathbf{ZL}}} \right) +\upmu _{k} \left( {{\mathbf{Z}} - {\mathbf{F}}_{k} + {\raise0.7ex\hbox{${{\mathbf{Y}}_{k}^{2} }$} \!\mathord{\left/ {\vphantom {{{\mathbf{Y}}_{k}^{2} } {\upmu _{k} }}}\right.\kern-\nulldelimiterspace} \!\lower0.7ex\hbox{${\upmu _{k} }$}}} \right)$$ + $$\upmu _{k} {\mathbf{X}}^{T} \left( {{\mathbf{XZ}} - {\mathbf{X}} + {\mathbf{G}}_{k} {\mathbf{X}} + {\mathbf{P}}_{k} - {{{\mathbf{Y}}_{k}^{1} } \mathord{\left/ {\vphantom {{{\mathbf{Y}}_{k}^{1} } {\upmu _{k} }}} \right. \kern-\nulldelimiterspace} {\upmu _{k} }}} \right)$$ and $$\upeta _{1} = \beta \left\| {\mathbf{L}} \right\|_{2} +\upmu _{k} \left( {1 + \left\| {\mathbf{X}} \right\|_{2}^{2} } \right).$$

**Updating G**

Similar to the solution of **Z**, the SVT operator is employed in computing **G**. The optimal solution ${\mathbf{G}}_{k + 1}^{*}$ is

20 $${\mathbf{G}}_{k + 1}^{*} = \Theta_{{{1 \mathord{\left/ {\vphantom {1 {\upeta _{2}\upmu _{k} }}} \right. \kern-\nulldelimiterspace} {\upeta _{2}\upmu _{k} }}}} \left( {{\mathbf{G}}_{k} - {{\nabla_{zq} \left( {{\mathbf{G}}_{k} } \right)} \mathord{\left/ {\vphantom {{\nabla_{zq} \left( {{\mathbf{G}}_{k} } \right)} {\upeta _{2} }}} \right. \kern-\nulldelimiterspace} {\upeta _{2} }}} \right),$$

where $$\nabla_{gq} \left( {{\mathbf{G}}_{k} } \right) =\upmu _{k} \left( {{\mathbf{XZ}}_{k + 1} - {\mathbf{X}} + {\mathbf{G}}_{k} {\mathbf{X}} + {\mathbf{P}}_{k} - {{{\mathbf{Y}}_{k}^{2} } \mathord{\left/ {\vphantom {{{\mathbf{Y}}_{k}^{2} } {\upmu _{k} }}} \right. \kern-\nulldelimiterspace} {\upmu _{k} }}} \right){\mathbf{X}}^{T}$$ and $$\upeta _{2} =\upmu _{k} \left\| {\mathbf{X}} \right\|_{2}^{2}$$.

**Updating F**

The below sub-problem *w.r.t.* **F** is

21 $$\begin{aligned} {\mathbf{F}}_{k + 1} &= \arg \mathop {\min }\limits_{{\mathbf{F}}} L\left( {{\mathbf{Z}}_{k + 1} , \, {\mathbf{G}}_{k + 1} , \, {\mathbf{F}}, \, {\mathbf{P}}_{k} , \,\upmu _{k} , \, {\mathbf{Y}}_{{_{k} }}^{1} , \, {\mathbf{Y}}_{{_{k} }}^{2} } \right) \\ {\kern 1pt} {\kern 1pt} &= \arg \mathop {\min }\limits_{{\mathbf{F}}} - {{\text{Tr}}}\left( {{\mathbf{A}}_{k + 1} {\mathbf{FB}}_{k + 1} } \right) + \left\langle {{\mathbf{Y}}_{{_{k} }}^{2} , \, {\mathbf{Z}}_{k + 1} - {\mathbf{F}}_{k} } \right\rangle {\kern 1pt} {\kern 1pt} { + }{{\upmu _{k} } \mathord{\left/ {\vphantom {{\upmu _{k} } 2}} \right. \kern-\nulldelimiterspace} 2}\left\| {{\mathbf{Z}}_{k + 1} - {\mathbf{F}}_{k} } \right\|_{F}^{2} . \\ \end{aligned}$$

Equation (21) is the smooth convex planning problem. Different to the solving rules of **Z** and **G**, we can differentiate Eq. (21) and set it to zero to gain the answer of **F**. Its optimal solution is

22 $${\mathbf{F}}_{k + 1} = {{{\mathbf{A}}_{k + 1} {\mathbf{B}}_{k + 1}^{T} } \mathord{\left/ {\vphantom {{{\mathbf{A}}_{k + 1} {\mathbf{B}}_{k + 1}^{T} } {\upmu _{k} }}} \right. \kern-\nulldelimiterspace} {\upmu _{k} }} + {\mathbf{Z}}_{k + 1} + {\mathbf{Y}}_{{_{k} }}^{2} .$$

**Updating P**

Calculating **P** has to optimize the following objective:

23 $$\begin{gathered} {\mathbf{P}}_{k + 1} = \arg \mathop {\min }\limits_{{\mathbf{P}}} L\left( {{\mathbf{Z}}_{k + 1} , \, {\mathbf{G}}_{k + 1} , \, {\mathbf{F}}_{k + 1} , \, {\mathbf{P}}, \,\upmu _{k} , \, {\mathbf{Y}}_{{_{k} }}^{1} , \, {\mathbf{Y}}_{{_{k} }}^{2} } \right) \\ = \arg \mathop {\min }\limits_{{\mathbf{P}}} \lambda \left\| {\mathbf{P}} \right\|_{1} {\kern 1pt} + {\raise0.7ex\hbox{${\upmu _{k} }$} \!\mathord{\left/ {\vphantom {{\upmu _{k} } 2}}\right.\kern-\nulldelimiterspace} \!\lower0.7ex\hbox{$2$}}\left\| {{\mathbf{P}} - \left( {{\mathbf{X}} - {\mathbf{XZ}}_{k + 1} - {\mathbf{G}}_{k + 1} {\mathbf{X}} + \frac{{{\mathbf{Y}}_{{_{k} }}^{1} }}{{\upmu _{k} }}} \right)} \right\|_{F}^{2} . \\ \end{gathered}$$

The optimal solution to the above sub-problem *w.r.t.* **P** can be formulated by

24 $${\mathbf{P}}_{k + 1} = S_{{{\lambda \mathord{\left/ {\vphantom {\lambda {\upmu _{k} }}} \right. \kern-\nulldelimiterspace} {\upmu _{k} }}}} \left( {{\mathbf{X}} - {\mathbf{XZ}}_{k + 1} - {\mathbf{G}}_{k + 1} {\mathbf{X}} + {{{\mathbf{Y}}_{{_{k} }}^{1} } \mathord{\left/ {\vphantom {{{\mathbf{Y}}_{{_{k} }}^{1} } {\upmu _{k} }}} \right. \kern-\nulldelimiterspace} {\upmu _{k} }}} \right).$$

$S_{{{\lambda \mathord{\left/ {\vphantom {\lambda {\upmu _{k} }}} \right. \kern-\nulldelimiterspace} {\upmu _{k} }}}} \left( \cdot \right)$ is the shrinkage operator defined as $S_{{{\lambda \mathord{\left/ {\vphantom {\lambda {\upmu _{k} }}} \right. \kern-\nulldelimiterspace} {\upmu _{k} }}}} \left( \cdot \right) = {\mathbf{U\Sigma }}_{{{\lambda \mathord{\left/ {\vphantom {\lambda {\upmu _{k} }}} \right. \kern-\nulldelimiterspace} {\upmu _{k} }}}} {\mathbf{V}}^{T}$, ${{\varvec{\Sigma}}}_{{{\lambda \mathord{\left/ {\vphantom {\lambda {\upmu _{k} }}} \right. \kern-\nulldelimiterspace} {\upmu _{k} }}}} = {{\text{diag}}}\left( {\max \left\{ {\sigma_{i} - {\lambda \mathord{\left/ {\vphantom {\lambda {\upmu _{k} }}} \right. \kern-\nulldelimiterspace} {\upmu _{k} }}} \right\},0} \right)$.

**Updating** $$\mu_{k}$$**,** $${\mathbf{Y}}_{{_{k} }}^{1}$$ **and** $${\mathbf{Y}}_{{_{k} }}^{2}$$

After computing the above variables, two Lagrange multipliers ${\mathbf{Y}}_{{_{k} }}^{1}$ and ${\mathbf{Y}}_{{_{k} }}^{2}$ are given by

25 $$\left\{ \begin{gathered} {\mathbf{Y}}_{{k + {1}}}^{1} = {\mathbf{Y}}_{k}^{1} +\upmu _{{k + {1}}} \left( {{\mathbf{X}} - {\mathbf{XZ}}_{k + 1} - {\mathbf{G}}_{k + 1} {\mathbf{X}} - {\mathbf{P}}_{k + 1} } \right), \hfill \\ {\mathbf{Y}}_{{k + {1}}}^{2} = {\mathbf{Y}}_{k}^{2} +\upmu _{{k + {1}}} \left( {{\mathbf{Z}}_{k + 1} - {\mathbf{F}}_{{k + {1}}} } \right). \hfill \\ \end{gathered} \right.$$

The iteration rule about $\upmu _{k}$ is

26 $$\begin{aligned} \mu _{{k + 1}} & = \min \left( {\mu _{{\max }} ,\;\rho _{k} \mu _{k} } \right). \\ \rho _{k} & = \left\{ {\begin{array}{*{20}c} {\rho _{0} ,} & {{\text{if}}\;\mu _{k} \cdot \max \left\{ {\begin{array}{*{20}c} {\eta _{1} \left\| {{\mathbf{Z}}_{k} - {\mathbf{Z}}_{{k + 1}} } \right\|,\eta _{2} \left\| {{\mathbf{G}}_{k} - {\mathbf{G}}_{{k + 1}} } \right\|} \\ {\left\| {{\mathbf{F}}_{k} - {\mathbf{F}}_{{k + 1}} } \right\|,\left\| {{\mathbf{P}}_{k} - {\mathbf{P}}_{{k + 1}} } \right\|} \\ \end{array} } \right\} \le \varepsilon _{2} ,} \\ {1,} & {{\text{otherwise}}{\text{.}}} \\ \end{array} } \right. \\ \end{aligned}$$

The detailed algorithm about TGLRR model is showed in Algorithm 1.
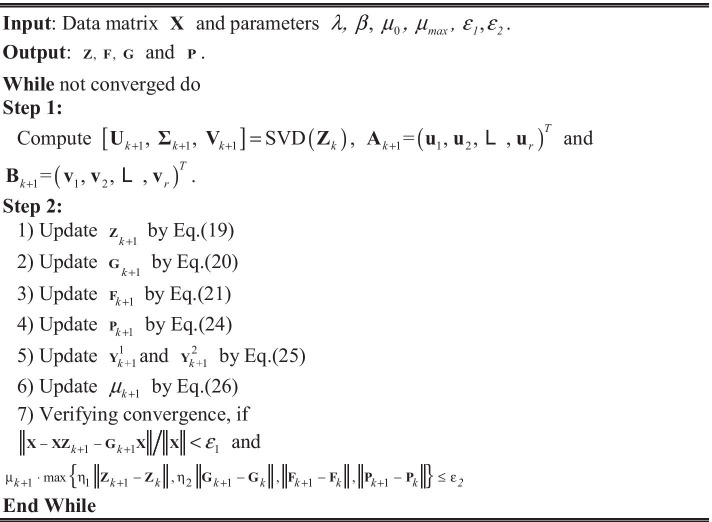


### Time complexity

In this subsection, the time complexity about TGLRR is discussed. Clearly, the main running time of TGLRR is expended on calculating the matrices **Z**, **F**, **G** and **P**. For the $m \times n$ input data matrix **X**, it has *m* genes and *n* samples. The time complexity of SVD method with respect to **Z** is ${\rm O}\left( {r_{{\mathbf{Z}}} n^{2} } \right)$ ($r_{{\mathbf{Z}}}$ is the lowest rank of **Z** decided by algorithm 1). For the same activity, the time complexity of SVD decomposition of **G** is ${\rm O}\left( {r_{{\mathbf{G}}} m^{2} } \right)$. The optimal solution of **F** can be obtained in ${\rm O}\left( {rmn} \right)$. In the resolving procedure of **P**, ${\mathbf{Y}}^{1}$ also needs to be updated. The computational cost of **P** and ${\mathbf{Y}}^{1}$ needs ${\rm O}\left( {mn^{2} + mr_{{\mathbf{P}}} n} \right)$ and ${\rm O}\left( {nm^{2} } \right)$, respectively. Since, $m > > n$ in our dataset, the total time cost of algorithm 1 is ${\rm O}\left( {nm^{2} } \right)$.

## Data Availability

The TCGA datasets that support the findings of this study are available in https://www.cancer.gov/about-nci/organization/ccg/research/structural-genomics/tcga. The results from the free trial version of the GeneCards (https://www.genecards.org/) did only be used for scientific research, but not be used for commercial purposes.

## Abbreviations

LRR: Low-Rank Representation
LADMAP: Linearized Alternating Direction with Adaptive Penalty
PCA: Principal Component Analysis
RPCA: Robust PCA
LE: Laplacian Eigenmap
LLE: Locally Linear Embedding
LLRR: Graph-Laplacian regularized LRR
LatLRR: Latent LRR
TNN: Truncated Nuclear Norm
TGLRR: Truncated nuclear norm and Graph-Laplacian regularized Low-Rank Representation
TCGA: The Cancer Genome Atlas
PAAD: Pancreatic Ductal Adenocarcinoma
ESCA: Esophageal Carcinoma
COAD: Colorectal Adenocarcinoma
CHOL: Cholangiocarcinoma
HNSC: Head and Neck Squamous Cell Carcinoma
ACC: Accuracy
NMI: Normalized Mutual Information
SVT: Singular Value Thresholding
SVD: Singular Value Decomposition
